# Supplementary material for: Efficient Synthesis and In Vitro Hypoglycemic Activity of Rare Apigenin Glycosylation Derivatives
Source: Molecules. 2023 Jan 5;28(2):533. doi: 10.3390/molecules28020533 (PMC9866095; doi:10.3390/molecules28020533)
Supplement: Supplementary file 1 [file molecules-28-00533-s001.zip › molecules-2096085-supplementary.pdf]

Supplementary Material

Efficient Synthesis and In Vitro Hypoglycemic Activity of Rare Apigenin Glycosylation Derivatives

Lin Zhao[#], Yuqiong Pei[#], Guoxin Zhang, Jiayao Li, Yujie Zhu, Mingjun Xia, Ke Yan, Wen Mu, Jing Han, Sen Zhang*, Jinao Duan*

Jiangsu Collaborative Innovation Center of Chinese Medicinal Resources Industrialization, Jiangsu Key Laboratory for High Technology Research of TCM Formulae, Nanjing University of Chinese Medicine, 138 Xianlin Road, Nanjing 210023, Jiangsu, China

[#] Lin Zhao and Yuqiong Pei contributed equally to this work.

* Corresponding author: Sen Zhang; Jinao Duan
E-mail address: zhangsci@njucm.edu.cn, zhangsci@163.com (S. Zhang); dja@njucm.edu.cn (J. Duan)

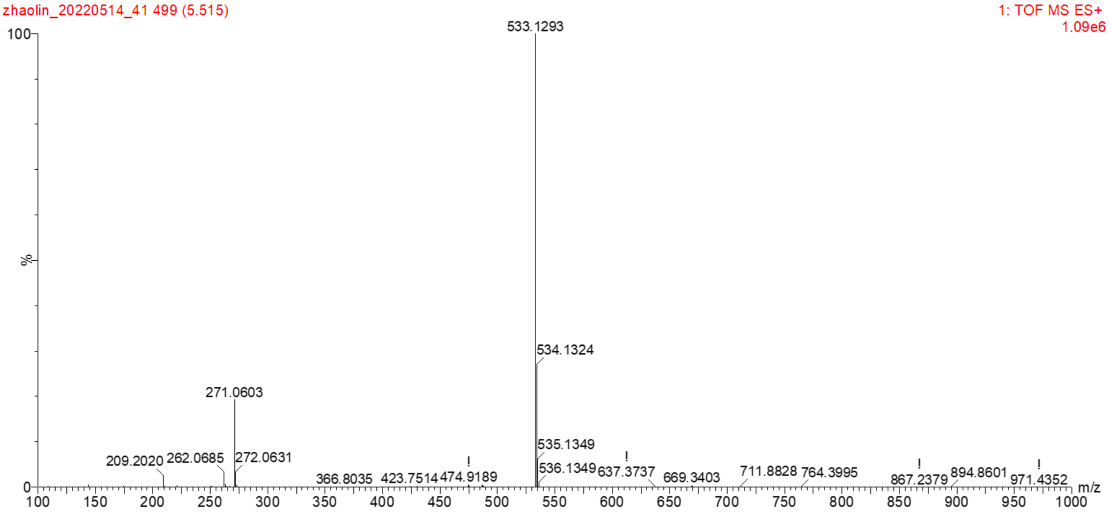

Figure S1 UPLC-QToF/MS analysis of SAG.

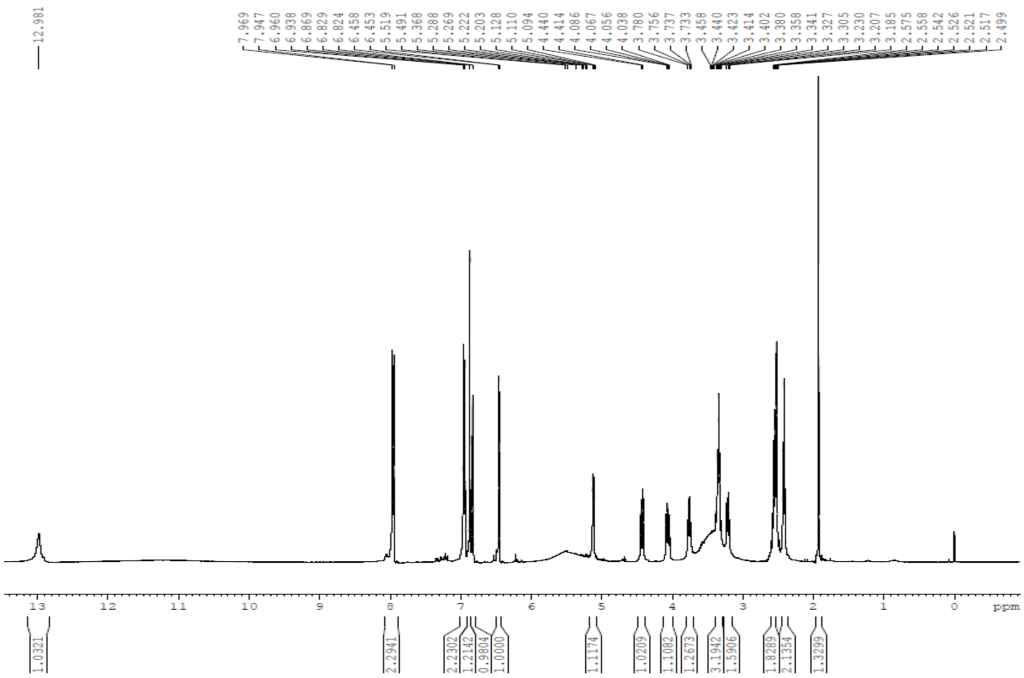

Figure S2 ^{1}H NMR spectrum of SAG.

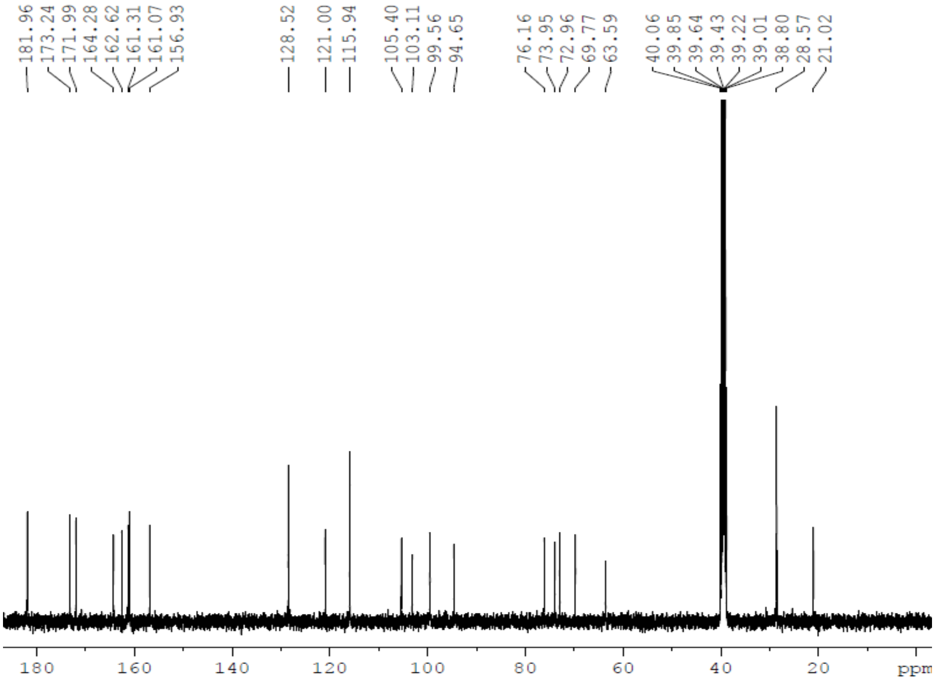

Figure S3 ^{13}C NMR spectrum of SAG.

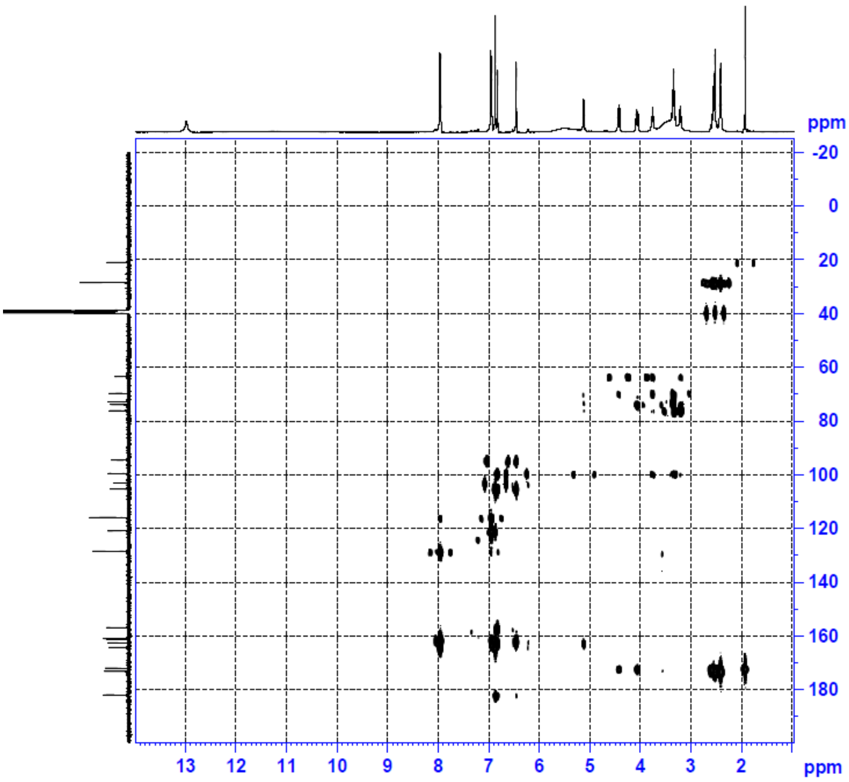

Figure S4 HMBC NMR spectrum of SAG.
